# Editorial: Heterogeneous Computing for AI and Big Data in High Energy Physics

**DOI:** 10.3389/fdata.2021.652881

**Published:** 2021-07-01

**Authors:** Daniele D’Agostino, Daniele Cesini

**Affiliations:** ^1^ IEIIT-National Research Council of Italy, Genoa, Italy; ^2^ CNAF-Italian Institute for Nuclear Physics, Bologna, Italy

**Keywords:** high performance computing, big data, artificial intelligence, high energy physics, heterogeneous computing

Heterogeneous computing denotes a scenario where different computing platforms are exploited for specific applications (Danovaro et al., 2014). While the demand for computational resources continues to grow with increasing need for querying and analyzing the volumes and rates of Big Data, energy efficiency is limiting the traditional approach to improve the compute capabilities of a data center by adding thousands of state-of-the-art x86 machines to an existing infrastructure in favor of adopting energy savvy devices (Cesini et al., 2017; D’Agostino et al., 2019). The result is that the computing nodes in data centers have different execution models, ranging from the traditional x68 architecture to GPUs, FPGAs (Papadimitriou et al., 2020) and other processor types like ARMs or more specialized processors as TPUs (Albrecht et al., 2019; Cass, 2019). For example, GPUs are used in many scientific applications based on regular domains and are delivering performance that is orders of magnitude better than traditional cores. They are also widely used in deep learning, especially the machine learning training phase. The FPGAs, being integrated circuits that can be configured by the programmer to implement a certain function, tries to close the gap between hardware and software.

Within this context the research topic collects five papers showing very interesting experiences in adopting heterogeneous architectures, for AI and Big Data applications in High Energy Physics.

In GPU-accelerated machine learning inference as a service for computing in neutrino experiments (Wang et al.) the authors discuss the performance achieved by exploiting GPU resources as a service for the ProtoDUNE-SP reconstruction chain developed in the context of the Deep Underground Neutrino Experiment (DUNE). This contribution represents one of the first experiences in which machine learning is acceleratedis accelerated with GPUs in neutrino software frameworks. The most time-consuming task, the track and particle shower hit identification, has been accelerated by a factor of 17.

In Heterogeneous reconstruction of tracks and primary vertices with the CMS pixel tracker (Bocci et al.) the authors describe an heterogeneous implementation of pixel tracks and vertices reconstruction chain on GPUs able to achieve high performance speedup values. The resulting framework has been developed for being integrated in the CMS particle detector reconstruction software, CMSSW (http://cms-sw.github.io), which is employed to detect particle and phenomena resulting from high-energy collisions in the LHC by the CMS experiment.

In Distance-Weighted Graph Neural Networks on FPGAs for Real-Time Particle Reconstruction in High Energy Physics (Iiyama et al.) the authors present a novel method to export graph neural networks from complex modern machine learning packages to an efficient FPGA implementation. The main characteristics is that this implementation is able to perform nontrivial physics tasks with a sub-microsecond latency, therefore it represents a suitable solution for low-latency applications, such as the Level-1 trigger stage of LHC.

In CLUE: A Fast Parallel Clustering Algorithm for High Granularity Calorimeters in High-Energy Physics (Rovere et al.) the authors propose a novel density-based clustering algorithm exploiting a new, highly parallelizable way of assigning points to clusters. Experiments have been performed on a synthetic dataset that resembles high occupancy events in high granularity calorimeters operated at HL-LHC with speedup values up to 112 on a GPU.

In Porting HEP Parameterized Calorimeter Simulation Code to GPUs (Leggett et al.) the authors describe an accelerated version of the ATLAS Calorimeter Fast-Simulation on GPUs. Several techniques have been incorporated the implementation to increase the GPU overall utilization, because the compute-to-I/O ratio is not very large, and the achieved results are interesting. A key topic of the work is represented by the introduction of performance portability through Kokkos, a C++ cross-device compatibility library. Authos discuss the different issues encountered during the porting process, the adopted solutions and present a comparison of the achievable performance with respect to previous approaches.
